# Effects of stocking at the parr stage on the reproductive fitness and genetic diversity of a wild population of Atlantic salmon (*Salmo salar* L.)

**DOI:** 10.1111/eva.13374

**Published:** 2022-04-18

**Authors:** Raphaël Bouchard, Kyle Wellband, Laurie Lecomte, Louis Bernatchez, Julien April

**Affiliations:** ^1^ Département de Biologie Université Laval Québec Quebec Canada; ^2^ Institut de Biologie Intégrative et des Systèmes (IBIS) Université Laval Québec Quebec Canada; ^3^ Direction de l’expertise sur la faune aquatique Ministère des Forêts, de la Faune et des Parcs du Québec Québec Quebec Canada

**Keywords:** captive breeding, genetic diversity, mature male parr, parentage assignment, supplementation

## Abstract

Captive‐breeding programs are among the most adopted conservation practices to mitigate the loss of biodiversity, including genetic diversity. However, both genetic and nongenetic changes occurring in captivity can reduce the fitness of supplemented individuals, which complicate rehabilitation efforts. In the case of Atlantic salmon, the intensity of changes that occur in captivity and their impact on fitness will vary with the stocking practice adopted. In this study, we test whether salmon stocked at the parr stage have reduced reproductive success compared with their wild conspecifics and whether they contribute to increase genetic diversity in the targeted population. To do so, we use high‐throughput microsatellite sequencing of 38 loci to accurately assign 2381 offspring to a comprehensive set of possible parents from a supplemented Atlantic salmon population in Québec, Canada. Captive‐bred salmon stocked at the parr stage had fewer mates than their wild conspecifics, as well as a reduced relative reproductive success (RSS) compared with their wild counterparts. Nonetheless, in comparison with previous studies, stocking at the parr stage significantly improved RSS compared with salmon stocked as smolts and they displayed a reduction in reproductive success similar to salmon stocked as fry, which spend less time in captivity than parr. Moreover, supplementation of captive‐bred salmon significantly contributed to increasing genetic diversity. These results should contribute to informing resource managers in determining the best stocking practice to enhance Atlantic salmon populations.

## 1 INTRODUCTION

As the number of critically endangered species increases, captive‐breeding programs have become important tools to preserve genetic diversity and fitness within a population (Fraser, 2007). However, the success of such programs depends on their ability to produce individuals with phenotypes that closely match one of wild individuals and that can contribute to population productivity (Frankham, 2008). Indeed, rearing a species in captivity can lead to unintentional selection for phenotypes that have a high fitness in captivity but low fitness in the wild. In turn, this can lead to domestication if not carefully managed (Frankham et al., 2008). Additionally, efforts to mitigate domestication selection in captivity can be hindered by phenotypic plasticity, which can also contribute to the phenotypic mismatch between captive‐reared individuals and their wild conspecifics (Christie et al., 2014; Johnsson et al., 2014; Lorenzen et al., 2012; Williams & Hoffman, 2009). Hence, both genetic and nongenetic effects occurring during captive rearing can complicate population supplementation or rehabilitation efforts.

Over the years, captive‐breeding programs have been implemented to conserve over 300 fish species by releasing billions of individuals reared in captivity in their native habitat (Brown & Day, 2002). Yet, many supplemented populations remain at historically low abundance level despite massive investment put into these programs (Fraser, 2007). This phenomenon is particularly well documented in salmonids, for which decades of studies have shown that captive‐bred individuals may experience reduced fitness in the wild compared with their wild conspecifics (e.g., Hagen et al., 2021; Hindar et al., 1991; Ryman & Laikre, 1991; Valiquette et al., 2014; Waples, 1991; Waples et al., 2016). For instance, in the case of steelhead trout, *Oncorhynchus mykiss* Walbaum, previous studies have demonstrated that even a few generations of captive rearing can induce genetic changes that reduce the reproductive fitness of captive‐bred individuals and their offspring in the wild (Araki, Cooper, et al., 2007; Araki, Waples, et al., 2007; Araki et al., 2008; Christie et al., 2012). Hence, while captive‐breeding programs can indeed prevent loss of genetic diversity in salmonids, their efficacy to supplement wild populations has remained contentious (Brown & Day, 2002; Fraser, 2007). Nonetheless, these programs are often the last standing battalion for resource managers to prevent the loss of populations at risk of imminent extinction such as in the case of native populations of Atlantic salmon (*Salmo salar*) (Snyder et al., 1996).

Atlantic salmon (*Salmo salar*) has been targeted by such programs for decades following a generalized decline in abundance across its range (ICES, 2019). As for other salmonids, rearing in captivity has been reported to induce changes in morphology, physiology, behavior, and life history traits that ultimately impact the fitness of released individuals (reviewed in Jonsson & Jonsson, 2009). However, the intensity of these changes and their impact on fitness will vary with the time spent in captivity. For instance, Milot et al. (2013) demonstrated that captive‐bred salmon that spent more time in captivity in early life experienced a greater reduction in reproductive success compared with their wild conspecifics. Hence, while salmon stocked at the fry stage had 71% of the reproductive success of their wild counterpart, salmon stocked as smolt only had 42% of their reproductive success.

While these results suggest that managers should limit the time spent in captivity to a minimum, the decision on which life stages to stock should represent a trade‐off between the gain of survival obtained in captive settings compared with the wild, the survival of captive‐bred individuals after being released in the wild, and the reduction in reproductive fitness experienced by the one that survives (Jonsson & Jonsson, 2009). To this end, stocking at the parr stage could represent a potential optimal trade‐off. Indeed, the parr stage is the one that experiences the least mortality in the wild compared with younger life stages (Cunjak & Therrien, 1998). Stocking at this stage would therefore maximize the gain of survival obtained in captive settings (Caron et al., 1999; Cunjak & Therrien, 1998). Moreover, the survival of released individuals after one summer is known to increase with the stage of development in Atlantic salmon (Coghlan & Ringler, 2004; Johnson et al., 2004). Hence, stocking parr would allow managers to benefit a significant increase in survival after being released compared with stocking of younger stages, which can make stocking efforts more cost‐effective (Jonsson & Jonsson, 2009). However, to our knowledge, no study documented the reproductive fitness of salmon stocked at this life stage. A reduced relative reproductive success (RSS) of released fish can eventually hinder population productivity, thus threatening rather than enhancing recipient populations (Chilcote et al., 2011; O’Sullivan et al., 2020). Therefore, documenting the reproductive success of salmon stocked as parr can provide a more comprehensive understanding of the impact of this stocking practice on managed populations. In turn, this information should guide resource managers’ decision on what best stocking practice to adopt for the restoration of Atlantic salmon populations.

Here, we test whether Atlantic salmon stocked at the parr stage have reduced reproductive success compared with their wild conspecifics. Our study system is a wild anadromous population spawning in the Rimouski River, Québec, Canada. This river has been targeted by several stocking programs since 1992, but the current program is now gradually ending since the population reached its appropriate conservation target from 2017 to 2020 (MFFP, 2021). Since this captive‐breeding program ultimately aimed to preserve genetic diversity in the targeted population, we further evaluated the contribution of captive‐bred salmon to increase the number of breeders (Nb) and allelic richness. To do so, we compared the relative contribution of captive‐bred salmon to the contribution of wild anadromous salmon and wild mature male parr, which become sexually mature at a size 200 times less than anadromous males.

## 2 MATERIALS AND METHODS

### 2.1 Study site

The Rimouski River is located on the south shore of the St. Lawrence Estuary, Québec, Canada (48°44′N; 68°53′W). On average, 723 adult Atlantic salmon spawners returned to the river annually from 2014 to 2018 (MFFP, 2020). A dam acting as a complete barrier to upstream migration is located four km upstream to the river mouth, at the level of a natural impassable waterfall. During their upstream migration, adult fish are trapped in a cage and transported 1 km above the dam to be released upstream. Spawning grounds and suitable habitats for Atlantic salmon juveniles extend over a stretch starting from the dam and ending 21 km upstream at a second impassable waterfall. Spawning also occurs downstream of the dam; this minor section of the river and the salmon that remained there are therefore not included in this study.

### 2.2 Captive‐breeding program of the Rimouski River

The captive‐breeding program of Atlantic salmon in the Rimouski R. started in 1992, and from 1992 to 2019, a total of 716,176 fry, 884,624 parr, and 297,019 smolts have been stocked. Starting in 2012, the broodstock was comprised of about 20 males and 20 females, with a semi‐factorial crossing design using a minimum of 3 females and 3 males. One third of the broodstock held in captivity was replaced on an annual basis, and no individual spawned more than three times. For this program, the number of juveniles to be stocked annually (about 60,000) was determined as detailed in Bernatchez (2009) in order to result in at least a 15% increase in abundance while limiting the effective population size reduction at less than 10%. Broodstock were collected from the Rimouski R. each year and crossed at the Québec Government hatchery located in Tadoussac. None of these captive breeders were born in the hatchery and kept all its life in captivity. However, some returning adult trapped at the dam and brought to the hatchery each year to maintain the pool of breeders may have been themselves offspring from captive‐born fish that had been released as fry, parr, or smolts in the Rimouski R. during past supportive breeding years, when stocked salmon were not marked. From 2012 to 2017, stocked salmon were young‐of‐the‐year weighting 3.21 to 3.54 g. Adipose fins were removed from stocked salmon for future identification purposes. An average of 14% of salmon returning in the Rimouski River from 2014 to 2018 was of hatchery origin.

### 2.3 Sampling

#### 2.3.1 Adult sampling and stocked fish identification

Measurements and fin samples (adipose or caudal) were taken on all anadromous adults in 2018 when fish were manipulated to release them above the dam. The genetic sex of every returning adult was determined using the King and Stevens (2019) PCR amplification‐based method. Adults were identified as hatchery born using the presence/absence of adipose fin, which was clipped on captive‐bred fish.

#### 2.3.2 Fry sampling

From July 15 to August 15, 2019, young‐of‐the‐year (or fry, age 0+) born in the river were sampled using electrofishing over a stretch spanning 21km from the dam to an impassable waterfall. The very small tributaries of this river stretch do not provide known spawning grounds or significant habitat for juveniles because of their limited accessibility. Fry samples were conserved in a 15‐ml Falcon tubes filled with 95% ethanol. Within every 1 km reach, we selected 12 electrofishing sites (mean area 100 m^2^) given their fry habitat suitability index, which was provided by the Québec Minister of Forests, Wildlife and Parks (MFFP). Each site was electrofished once to maximize the amount of site sampled in a single day. To assess the effect of the number of offspring sampled on the potential for detection of anadromous parents, we subsampled 50 to 2381 offspring by steps of 50, with each step being subsampled 1000 times. In the case where enough offspring were sampled, the number of identified parents should reach a plateau (Figure S1).

### 2.4 Molecular analyses

DNA was extracted from adult's adipose fin tissue and fry's caudal fin tissue using the salt extraction method described by Aljanabi and Martinez (1997). Fifty‐two microsatellite loci were then amplified by PCR in two multiplexes developed by Bradbury et al. (2018) (their panels 1a and 2a). Locus amplification followed the protocol of Zhan et al. (2017); two sets of polymerase chain reactions (PCRs) were used, a multiplex PCR and an index PCR. Each multiplex PCR was performed using Qiagen Multiplex Master Mix. Each oligonucleotide in the multiplex reaction was tailed with Illumina sequencing primer sequence and served as oligo‐binding sites in the subsequent index PCR. PCR multiplex conditions were the same for the two multiplexes and included 5 µl Qiagen Multiplex Master Mix, 10 ng DNA, and 2.4 µl Oligo Mix for a total volume of 25 µl. Thermal reaction conditions included 95°C, 25X (94°C for 30 s, 65°C for 3 m, and 72°C for 30 s), and 72°C for 30 s. Multiplex PCR products were pooled (per sample) in equal volume amounts, cleaned using Quanta Bio SparQ PureMag Beads, and then used as a template for the index PCR. Libraries were sequenced at 10–12 pM concentration at the genomic platform of the Institut de Biologie Intégrative et des Systèmes (IBIS), Université Laval, Québec (http://www.ibis.ulaval.ca/). Sequencing was performed using Illumina MiSeq (Illumina) and the MiSeq Reagent Kit V3 with 150 cycles in one direction and dual indexing. Indexed individuals were demultiplexed with the MiSeq Sequence Analysis software. The allelic sizes were then scored using MEGASAT (Zhan et al., 2017) setting minimum depth (per sample per locus) at 20 reads; that is, alleles with less than 20 reads were not called. Examination of histogram outputs (depth vs. allele size) from MEGASAT confirmed allele scores, and we adjusted scores when necessary. Loci with more than 10% missing data were removed from the dataset to ensure precision to our parentage analysis.

### 2.5 Parental allocation of Fry

Parentage allocation was conducted using Cervus v 3.0 (Kalinowski et al., 2007; Marshall et al., 1998) and Colony2 (Jones & Wang, 2010) that differ in their approach to parentage assignment. Cervus3 uses simulated parents and offspring to determine cut‐off points of log‐likelihood (LOD) scores for true parents, which are then used to identify parent–offspring pairs in empirical data. Cervus3 was first used to find the most probable mother–offspring and father–offspring dyad. As mentioned above, Rimouski R. is known for harboring mature male parr. Those males were not sampled in 2018; thus, the full‐likelihood approach implemented in Colony2 (Jones & Wang, 2010) was used to infer their genotypes from the pedigree analysis. Briefly, Colony2 uses a groupwise method to find the most likely configuration of full‐sib and half‐sib families in the data. Since all anadromous fish (female and male) were transported above the dam, any missing genotypes must belong to mature male parr. Therefore, grouping offspring into full‐sib and half‐sib families allowed us to infer the genotype of mature male parr given the genotype of the mother when the offspring was not already assigned to an anadromous father. Wang (2004) has previously tested the impact of accounting for genotyping error in parentage analysis using the Colony algorithm. Using simulations, he has shown that it increased the accuracy of assignment from 60% to 100% using a wide range of possible typing error rates (~0.001%–0.40%). To increase our confidence in inferred male genotypes, we used a previous estimate of genotyping error of 0.14% for the microsatellite panel developed by Bradbury et al. (2018) to provide as input in Colony2.

### 2.6 Detection of first‐generation (*F*_0_) migrant

Mobley et al. (2018) showed that local Atlantic salmon exhibit higher fitness in their native river compared with dispersers straying from other populations. Therefore, we conducted a first‐generation (*F*_0_) migrant detection analysis with Geneclass2 (Piry et al., 2004) to remove those potential dispersers from statistical analysis pertaining to the comparison of reproductive success between wild and captive‐bred salmon. Here, local salmon refers to adult salmon that were born in the Rimouski River and dispersers were salmon that were born in another river but that reproduced in the Rimouski River. All adult salmon were included in this analysis (1 sea‐winter and multi‐sea‐winter salmon). Since we had no genotypic reference of potential source populations with this microsatellite panel, we computed the likelihood of individual genotypes within the Rimouski population (L_home). To do so, we used the frequency‐based method of Paetkau et al. (1995) and the Monte Carlo resampling algorithm developed by Paetkau et al. (2004) simulating 10,000 individuals to estimate the probability of an observed multi‐locus genotype. Individuals that had a probability below 0.01 (type I error) were considered as *F*_0_ migrants and thus removed from downstream analyses.

### 2.7 Analysis of relative reproductive success of captive‐bred salmon

We estimated relative fitness of hatchery‐born fish to that of wild‐born fish. To do so, we used the number of offspring assigned to a given spawner as a measure of reproductive fitness. We do not expect differences in number of offspring sampled between captive‐bred and wild salmon to be due to different habitat use since previous studies did not find such differences (Einum & Fleming, 2001). This measure is necessarily partial because not all offspring of a given spawner were collected. Then, we computed the ratio of reproductive fitness of the hatchery/wild fish, which gives a measure of relative reproductive success (RRS) using eq. 14 of Araki and Blouin (2005):

$${\text{RRS}}_{\left[\text{unbiased}\right]}=\frac{\hat{W_{x}}‐\left(\frac{N_{\text{offspring}}‐N_{\text{assigned}}}{N_{\text{parent}}}\right)\left(\frac{\hat{b}}{1‐\hat{b}}\right)}{\hat{W_{y}}‐\left(\frac{N_{\text{offspring}\;}‐N_{\text{assigned}}}{N_{\text{parent}}}\right)\left(\frac{\hat{b}}{1‐\hat{b}}\right)}$$

where $\hat{W_{x}}$ and $\hat{W_{y}}$ are the mean fitness of individuals in each group, $N_{\text{offspring}}$ is the total number of offspring sampled, $N_{\text{assigned}}$ is the number of offspring assigned to a parent, $N_{\text{parent}}$ is the total number of parents, and $\hat{b}$ is the rate that an offspring, which is not assigned to its true parent, is assigned to an untrue parent. RRS calculations were conducted independently for single‐sea‐winter (1SW) males, and multi‐sea‐winter (MSW) males and females. Analyses were performed separately for each sex since they may respond differently to the hatchery environment (Christie et al., 2014). To test for statistical significance, we perform nonparametric two‐tailed permutations to test the hypothesis that captive‐bred individuals have lower fitness than that of wild (i.e., whether RRS < 1.0). Briefly, numbers of offspring assigned to each parent were permutated 1,000,000 times (without replacement) and the probability of obtaining a value equal or larger than the observed fitness difference was evaluated (see Araki & Blouin, 2005 for details).

Following Araki and Blouin (2005), we used the maximum‐likelihood method developed by Kalinowski and Taper (2005) to calculate confidence intervals for RRS estimates. Their method is appropriate for our study because it assumes that the frequencies of hatchery and wild fish are known exactly and that the only uncertainty in the estimate of RRS comes from sampling a finite number of offspring. Considering this set of assumptions, maximizing the number of offspring sampled allowed us to obtain more precise estimates of the realized RRS regardless of the adult sample size.

### 2.8 Effects of captive breeding on components of reproductive fitness

We assessed the captive‐breeding effects on the number of offspring assigned and the number of mates. The main idea was to evaluate which of the two components of reproductive fitness was significantly different between captive‐bred and wild salmon. The number of mates was inferred from the sample of offspring and was used as a proxy for mating success. For males, mating success is determined by a combination of fertilization opportunities, relative fertilization success during a spawning event, and the viability of their offspring, which depends on genetic “quality” and maternal effects (egg size, nest site, guarding, etc.) (Petersson & Järvi, 2015). On the contrary, female mating success is determined by fecundity, the number of eggs successfully deposited, nest characteristics, other maternal effects, and the genetic quality of her mates, over which she has little control (Petersson & Järvi, 2015). We performed this analysis separately for females and males using a zero‐inflated negative binomial model (ZINB). We applied a ZINB model because estimates of the number of offspring assigned and number of mates contained a high proportion of individuals without any offspring, which hampers the possibility to identify their mates. Zero‐inflated mixture models consisted of a binomial model for the frequency of zeros and, conditional on this, a count model using a negative binomial distribution. For females, we performed individual analysis per mate type (mature male parr, 1SW, and MSW males) and an analysis combining all three mate types. For each model explaining the number of offspring assigned to females, we built a global ZINB model using origin (i.e., captive‐bred/wild), fork length, and number of mates (Table S2). For the analysis pertaining to the number of mates, we built a global ZINB model using origin and fork length. To analyze the factors influencing the number of offspring assigned to males, we built a global ZINB model using origin, number of years spent at sea (one (1SW) or more (MSW)), and number of mates and the interaction between time at sea and origin and between time at sea and number of mates. For the analysis pertaining to the number of mates for males, we built a global ZINB model using origin, time spent at sea, and the number of mates and the interaction between time at sea and origin.

For all models, we assessed the fit of the global model (model that contains all variables) by visualizing quantile–quantile residual distribution and rootogram, which graphically compares empirical frequencies with fitted frequencies from a given probability model with the package countreg in R (Zeileis & Kleiber, 2018; Figures S3 and S4). Then, we built a set of models made of all models nested within the global model (i.e., all combinations of including or excluding each variable) plus a null model (intercept only). Those models were ranked according to their AICc (a corrected measure of AIC for small samples), and ∆AICc (AICc of the model minus the AICc of the best model) was computed for each model. Following this procedure, we built a confidence set of models with all models with a ∆AICc < 4 (according to Hurvich & Tsai, 1989). Finally, we quantified the effect of variables appearing in the top models with multi‐model inference using the shrinkage method (Burnham & Anderson, 2002). In the shrinkage method, a parameter estimate (and error) of zero is substituted in models where the given parameter is absent, and the parameter estimate is obtained by averaging all models in the top model set. Hence, the zero method decreases the effect sizes (and errors) of predictors that only appear in models with small model weights. Every model was built using the “pscl” package in R 3.4.4.

### 2.9 Effect of captive‐bred fish on the effective Nb

To investigate the effect of captive‐bred fish on the effective Nb, we estimated Nb for three different datasets of fry corresponding to three different groups of parents: (i) wild anadromous breeders (*n* = 916), (ii) wild and captive‐bred anadromous breeders (*n* = 1560), and (iii) wild anadromous breeders and mature male parr (*n* = 1544). To do so, we used the LDNe program (Waples & Do, 2010) to estimate Nb and the contribution of wild and captive‐bred fish to Nb. We used a threshold of 0.05 as the lowest allele frequency that gives the least biased results according to Waples and Do (2010). Since the sample of parent‐fry assignment to wild breeders only was smaller than that also including the contribution of mature male parr, we subsampled these latter datasets using the same amount of fry as that of the wild breeder dataset. In this way, the threshold on the lowest allele frequency has the same effect on each dataset. Hence, we subsampled the parent‐fry assignment of datasets (ii) and (iii) 1000 times and calculated Nb on each subsampling step. Finally, we compared the value of Nb obtained in group (i) with the distribution generated with groups (ii) and (iii).

### 2.10 Effect of mature male parr and captive‐bred salmon on offspring's genetic diversity

To contrast the relative contribution of mature male parr in terms of allelic richness to that of captive‐bred males, we compared the total number of alleles across all loci found among progeny assigned to (i) wild anadromous pairs, (ii) wild and captive‐bred anadromous pairs and (iii) wild anadromous female and mature male parr for an increasing number of offspring sampled. We subsampled from 50 to 1500 fry, by steps of 50, 1000 times for each step, and estimated the total number of alleles found among fry assigned to the aforementioned groups of parents. We represented the differences in the number of alleles among these three groups with a Loess regression of the mean value of the total number of alleles found for each number of fry sampled considered, as well as the 95% distribution of values.

## 3 RESULTS

### 3.1 Adult population of the Rimouski River

From June 15 and October 30, 2018, 475 anadromous Atlantic salmon (273 males and 202 females) were transported above the dam, of which 26% were from the captive‐breeding program (*n* = 126) (Table 1). The anadromous population was composed of 57% (*n* = 271) salmon >63 cm, which are considered as MSW and of 43% (*n* = 204) salmon <63 cm, which were considered 1SW salmon (MFFP, 2016; MFFP, 2021). The proportion of MSW and 1SW was significantly different between wild and captive‐bred salmon ($\chi^{2}$ = 7.58, *p*‐value = 0.005) with the former having 186 (53%) MSW for 160 (45%) 1SW and the latter having 85 (67%) MSW for 41 (33%) 1SW. The ratio of 1SW to MSW males differed significantly between captive‐bred and wild males (captive‐bred: 40 1SW for 50 MSW, wild: 154 1SW for 51 MSW; X‐squared = 29.543, *p*‐value = <0.001). In total, Colony 2 estimated that 432 mature male parr fathered 750 offspring with a mean reproductive success of 1.69 offspring per mature parr that reproduced in our system. The presence of mature parr highly skewed the sex ratio, which was of 1 female to 1.43 male before accounting for them and of 1 female to 3.7 males when accounting for them. Eight individuals were identified as first‐generation disperser from populations using Geneclass2, of which two were MSW females and six were SSW males. Those individuals were removed from downstream analysis.

**TABLE 1:** Details of the genotyped adults and juveniles Atlantic salmon. The samples include all the returning adults caught at the Rimouski River dam from summer 2018 and the juveniles caught on the Rimouski River spawning grounds during spring 2019

	Female	Male	Total
Adult transported above dam	207	273	475
Adult used for assignments^a^	198	271	468
Born in the river (wild origin)	142	205	349
Born in the Rimouski hatchery	56	68	126
Returning as SSW	10	194	204
Returning as MSW	188	79	271
Fry assigned	2495	1617	2495

^a^ Difference between the number of adults used for assignment and the number transported above the dam is due to fish with missing or partial genotypes.

### 3.2 Parental allocation

After filtering for loci with more than 10% missing data on both parents and offspring dataset, 38 polymorphic microsatellite loci were retained for subsequent analyses. The number of alleles ranged from 3 to 13 per locus (average = 7), and observed heterozygosity ranged from 0.024 to 0.8396 with an average of 0.55 (Table S1). Average *F*_IS_ was −0.005 (95**%** CI**:** −0.01–0.0091), thus indicating the absence of the within‐river Wahlund effect (Table S1).

For the 475 adults transported above the dam, we obtained a complete genotype (38 out of 38 loci genotyped) for 468 individuals, which was then used for parental allocation (Table 1). A total of 2495 fry were sampled during July and August in 2019, genotyped, and assigned to putative parents that spawned above the dam during fall 2018. We assigned with probability >0.8 the paternity of 1617 and the maternity of 2495 offspring samples to anadromous adults and the paternity of 750 offspring to mature parr. The remaining 128 fry were assigned to mature parr with a probability lower than 0.8 and were thus excluded from subsequent analyses. Assignment probability was below 0.8 for those mature male parr because of missing data and smaller family sizes.

### 3.3 Description of the mating system

The reproductive success of females ranged from 0 to 67 (mean = 5.6, variance = 76.3; Figure S2) when they mated with mature male parr, from 0 to 29 (mean = 3.5, variance = 22.7) when they mated with 1SW males, and from 0 to 33 (mean = 4.4, variance = 33.5) when they mated with MSW males. For males, the number of inferred offspring ranged from 0 to 23 (mean = 3.7, variance = 21.9; Figure S2) for 1SW salmon and from 0 to 56 (mean = 11.6, variance = 173) for MSW salmon.

The total number of mates per female ranged from 0 to 33 (mean = 7, variance = 38.0; Figure S2). Mature male parr mates per female ranged from 0 to 26 (mean = 3.6, variance = 16.9), 1SW mates per female ranged from 0 to 7 (mean = 1.6, variance = 2.8), and MSW mates per female ranged from 0 to 8 (mean = 1.6, variance = 3.0). Based on parental allocation, males were also polygamous possibly to a lesser extent than females since we found offspring from a single mate female for 57 males of which 47 were 1SW males (13 captive‐bred and 35 wild; Figure S2). The number of mates for 1SW males ranged from 0 to 7 (mean = 1.64, variance = 2.43), and for MSW, it ranged from 0 to 16 (mean = 4.2, variance = 16.9).

### 3.4 Relative reproductive success of captive‐bred versus wild Atlantic salmon

**FIGURE 1:**
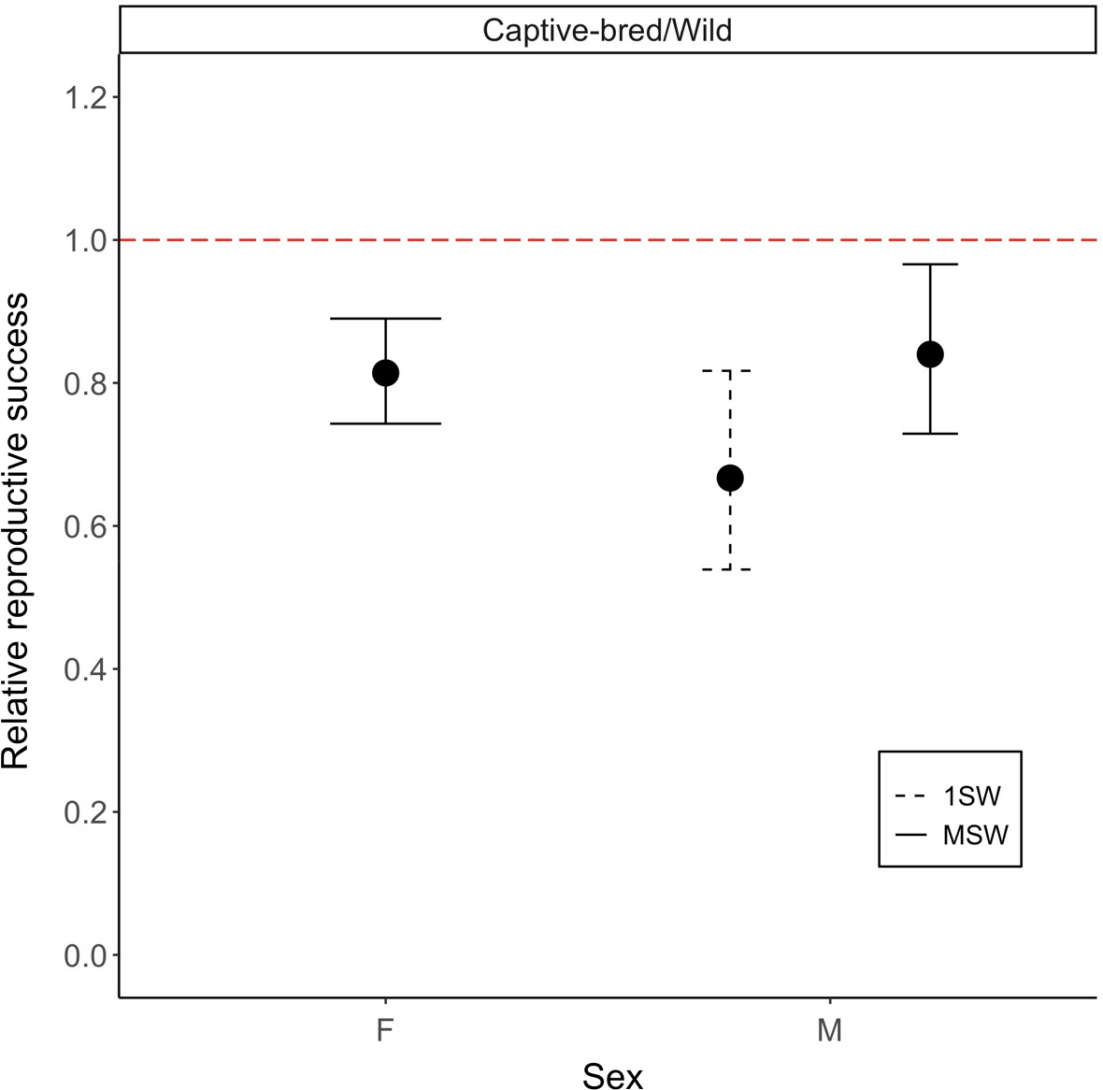
Maximum‐likelihood estimates of relative reproductive success (RRS) and their associated 95% confidence intervals for captive‐bred vs. wild Atlantic salmon. If captive‐bred and wild salmon had equal fitness, then RRS would be equal to 1 (dashed red line)

Estimates of RRS for captive‐bred versus wild salmon are shown separately for males and females and time spent at sea (1SW, MSW) in Table 2. Our results show that captive‐bred salmon had a reduced RRS compared with their wild counterparts. Thus, the RRS of MSW hatchery‐reared females was 0.805; this value was significantly lower than one (*p* < 0.01) based on both our permutation test and our maximum‐likelihood confidence interval estimation (Figure 1). For MSW hatchery‐reared males, RRS was 0.828; likewise, this value was significantly lower than one (*p* < 0.01) given both our permutation test and our maximum‐likelihood confidence interval estimation (Figure 1). The RRS of captive‐bred 1SW males was 0.649, which again was significantly lower than one (*p* < 0.01) compared with wild‐born 1SW males.

**TABLE 2:** Relative reproductive success (RRS) of naturally spawning *F*_1_ parent

Sex/winter at sea	*nF*_1_ (C‐B/W)	*nF*_2_ (H/W)	RS captive‐bred	Variance captive‐bred	RS wild	Variance wild	RRS	*p*‐value	80%/95% power
Female MSW	53/133	607/1860	11.5	101	14.0	238	0.805	<0.01	0.944/0.916
Male MSW	28/51	286/620	10.2	160	12.2	183	0.828	<0.01	0.913/0.870
Male 1SW	40/146	105/593	2.62	11.8	4.06	24.3	0.649	<0.01	0.885/0.829

Note

*nF*1 is the sample size for naturally spawning captive‐bred (C‐B) and wild (W) parents; *nF*2 is the number of offspring assigned to each group of parents. RS is the reproductive success measured as the mean number of offspring assigned per parent. Variance is the average of the squared differences from the mean reproductive success. RRS is calculated as the RS of captive‐bred fish over the RS of wild‐origin fish; associated *p*‐values are based on two‐tailed permutation tests. Statistical power is the RRS value that would be significant with 80% and 95% probability.

### 3.5 Effect of origin and male reproductive tactics on components of reproductive success

Prior to running ZINB, we used exploratory data analyses to determine the distribution of the number of offspring and number of mates within our dataset. The number of offspring and mates revealed a zero‐inflated negative binomial distribution with most individuals producing zero offspring, a trend that was similar for females and males (Figure S2).

**FIGURE 2:**
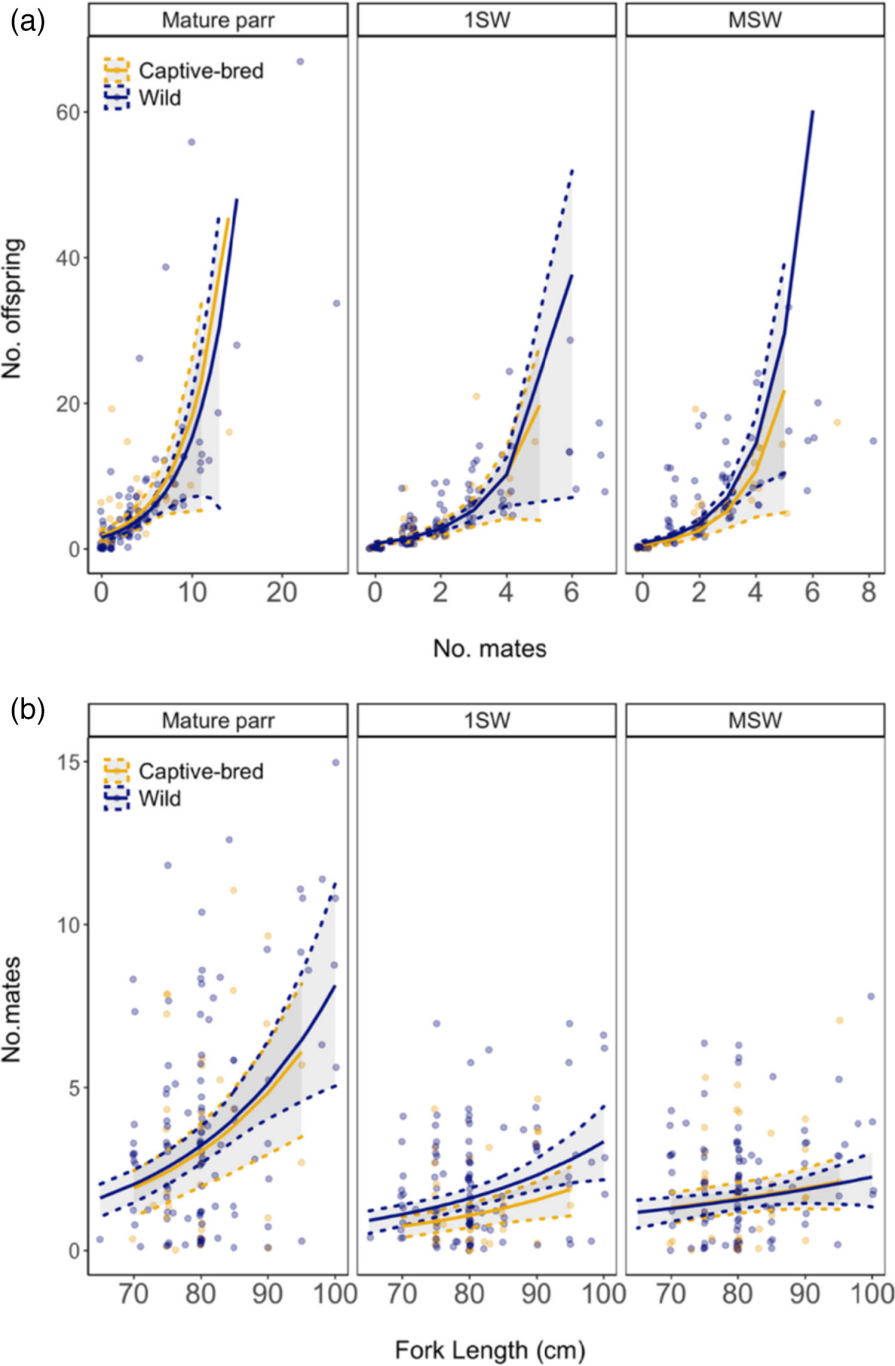
(a) Relationship between the number of offspring (No. offspring) and number of mates (No. mates) for female Atlantic salmon for three alternative reproductive tactics (mature parr, 1SW, and MSW). Colored lines represent ZINB model prediction for captive‐bred (blue) and wild (yellow) females, and gray areas and hatched lines represent 95% confidence intervals (CI) obtained by bootstrap. Circles show individual data points. (b) Relationship between number of mates (No. mates) and length of female Atlantic salmon for three alternative reproductive tactics (mature parr, 1SW, and MSW). Colored lines represent ZINB model prediction for captive‐bred (blue) and wild (yellow) females; gray areas and hatched lines represent 95% confidence intervals (CIs) obtained by bootstrap. Circles show individual data points

The number of offspring assigned to females increased with the number of mates for all three male alternative life history tactics (Tables 3 and 4; Figure 2a). However, females increased their reproductive success at a lower rate when they mated with mature male parr than when they mated with 1SW and MSW mates. Female length had a significant positive effect on the number of offspring assigned only when mating with MSW males. The number of offspring assigned to wild and captive‐bred females did not differ significantly. Females’ number of mates showed a positive relationship with length for all three types of male alternative life history tactics (Figure 2b). There was no difference between the number of mature male parr and MSW mates between captive‐bred females and wild females. However, captive‐bred females had significantly less 1SW mates than wild females.

**TABLE 3:** Summaries of ZINB model testing the effect of number of mates, length, and origin (wild/captive‐bred) and the effect of length and origin on the number of offspring assigned to females and number of mates, respectively

Parameter	Estimate ± SE	*z‐score*	*p*‐Value
Number of offspring			
Intercept	1.67 ± 0.36	4.58	<0.01
Number of mates	0.13 ± 0.01	10.88	<0.01
Length	−0.005 ± 0.007	0.84	0.358
Origin (Wild)	−0.05 ± 0.10	0.571	0.568
Zero inflation	−1.72 ± 0.25	6.89	<0.01
Number of mates			
Intercept	−0.76 ± 0.47	−1.61	0.109
Length	0.03 ± 0.01	6.07	<0.01
Origin (wild)	0.10 ± 0.10	0.10	0.319
Zero inflation	−1.29 ± 0.18	−7.12	<0.01

Note

The “zero inflation” term accounts for the large number of adults with zero reproductive success and number of mates in our sample.

**TABLE 4:** Summaries of ZINB model testing the effect of number of mates, length, and origin (wild/captive‐bred) and the effect of length and origin on the number of offspring assigned to females and number of mates, respectively, based on male alternative life history strategies

Parameter	Mature parr			1SW			MSW		
	Estimate ± SE	*z‐*score	*p*‐Value	Estimate ± SE	*z*‐score	*p*‐Value	Estimate ± SE	*z*‐score	*p*‐Value
Number of offspring									
Number of mates	0.24 ± 0.02	10.658	<0.01	0.67 ± 0.05	12	<0.01	0.69 ± 0.06	−11.789	<0.01
Length	−0.18 ± 0.16	−1.11	0.267	−0.027 ± 0.167	−1.075	0.282	0.03 ± 0.01	2.349	0.019
Origin (Wild)	−0.02 ± 0.01	−1.85	0.063	0.02 ± 0.17	−0.165	0.869	0.30 ± 0.17	1.716	0.086
Zero Inflation	−12.10 ± 90.06	−0.134	0.893	−12.97 ± 84.27	−0.154	0.878	−11.43 ± 43.51	−0.263	0.793
Number of mates									
Length	0.046 ± 0.05	9.410	<0.01	0.037 ± 0.009	4.053	<0.01	0.0187 ± 0.08	2.256	0.024
Origin (Wild)	0.057 ± 0.102	0.561	0.575	0.396 ± 0.180	2.204	0.028	−0.0243 ± 0.157	−0.154	0.878
Zero Inflation	−0.989 ± 0.179	−5.52	<0.01	−1.56 ± 0.45	−3.464	<0.01	−0.784 ± 0.204	−3.838	<0.01

Note

The “zero inflation” term accounts for the large number of adults with zero reproductive success and number of mates in our sample.

**FIGURE 3:**
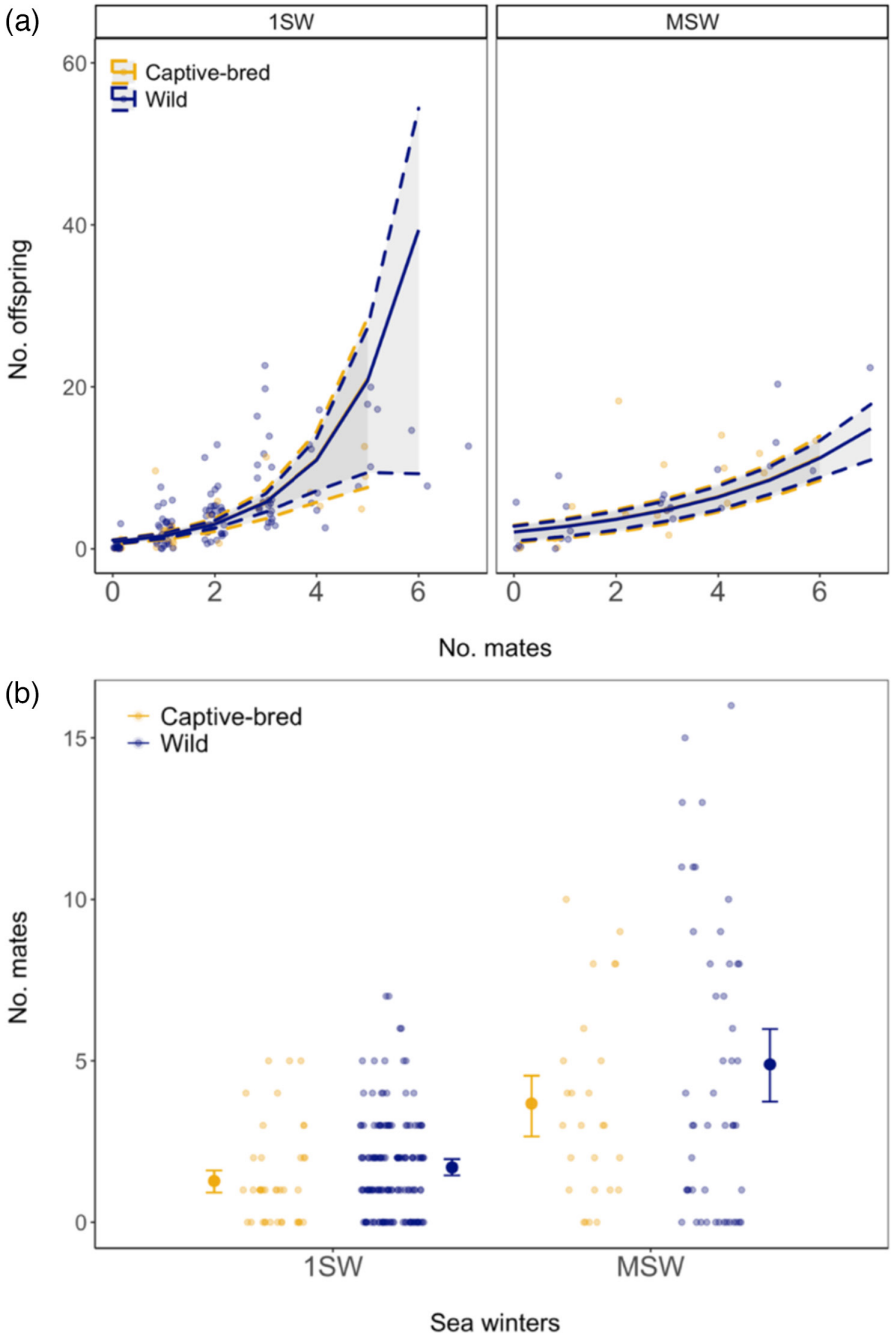
(a) Relationship between number of offspring (No. offspring) and number of mates (No. mates) for 1SW and MSW male Atlantic salmon. Lines represent ZINB model prediction for captive‐bred (blue) and wild (yellow) 1SW and MSW males; gray areas represent 95% confidence intervals (CI) obtained by bootstrap. (b) Relationship between number of mates (No. mates) and winters at sea (Sea winters) in male Atlantic salmon. Large circles with error bars represent the model prediction ±95% confidence interval (CI) obtained by bootstrap, while small circles show individual data points. Captive‐bred males had significantly less mates than wild males (*p* < 0.038)

MSW males had a higher number of offspring assigned than the 1SW salmon (Table 5, Figure 3a). Both 1SW and MSW males increased their number of offspring by mating with multiple females. However, 1SW males increased their reproductive success at a greater rate when mating with multiple females than MSW males. As for captive‐bred females, captive‐bred males did not show a significant reduction in offspring assigned relative to their wild counterparts in our ZINB model. 1SW males had fewer mating partners than MSW males, and both 1SW and MSW captive‐bred males showed a significant reduction in their number of mates compared with their wild counterparts (Figure 3b).

**TABLE 5:** Summaries of ZINB model testing the effect of number of mates, sea age, and origin (wild/captive‐bred) and the effect of sea age and origin on the number of offspring and number of mates, respectively

Parameter	Estimate ± SE	*z*‐score	*p*‐Value
Number of offspring			
Intercept	0.74 ± 0.18	4.23	<0.01
Number of mates	0.28 ± 0.03	10.59	<0.01
Sea age (1SW)	−0.90 ± 0.21	−4.37	<0.01
Sea age (1SW): number of mates	0.36 ± 0.06	6.18	<0.01
Origin (wild)	−0.01 ± 0.13	−0.08	0.93
Zero inflation	−12.27 ± 78.35	−0.157	0.876
Number of mates			
Intercept	1.44 ± 0.13	10.97	<0.01
Sea age (SSW)	−1.06 ± 0.12	−8.67	<0.01
Origin (wild)	0.28 ± 0.14	2.08	0.038
Zero inflation	−1.90 ± 0.33	−5.79	<0.01

Note

The “zero inflation” term accounts for the large number of adults with zero reproductive success and number of mates in our sample.

### 3.6 Estimation of the effect of captive‐bred anadromous salmon and of mature male parr on genetic diversity of offspring

**FIGURE 4:**
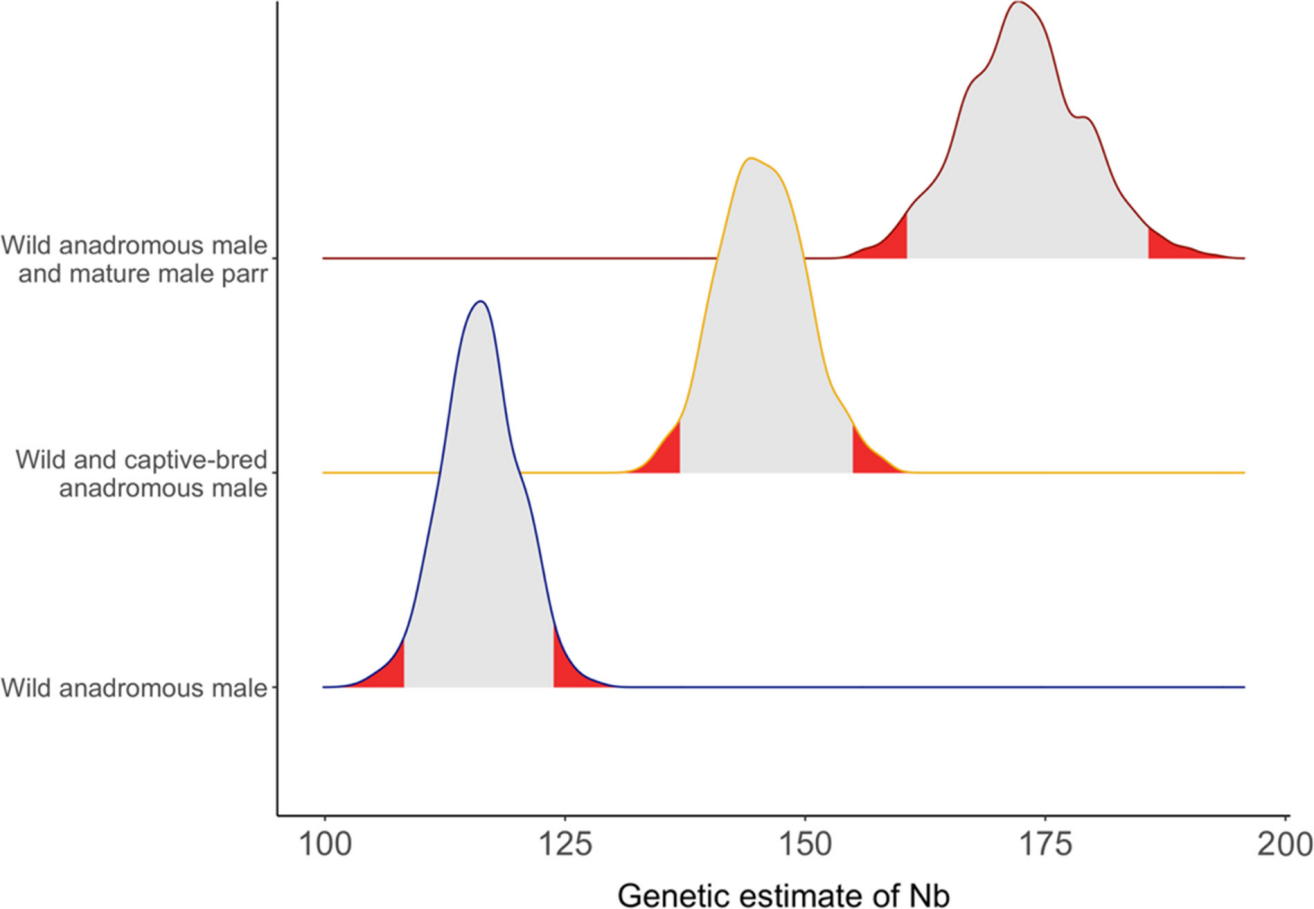
Distribution of genetic (LDNe) estimates of effective number of breeders (Nb) considering either only wild anadromous salmon, both wild and captive‐bred anadromous salmon or both wild anadromous salmon and mature male parr. The distribution of Nb calculated for wild anadromous salmon only was obtained directly from NeEstimator 2 (Do et al., 2014), whereas the distribution for wild and captive‐bred anadromous salmon or wild anadromous salmon and mature male parr was obtained by subsampling 916 fry from the parent‐fry assignment of wild and hatchery breeders 1000 times and calculating Nb on each subsampling step. The 2.5% and 97.5% tails of the distribution are indicated in red

Using LDNe to estimate Nb from the genetic data, we obtained values of 114 (CI: 110.8–121.7) in a scenario with only wild anadromous salmon contributing to reproduction of 146 (CI: 136.8–154.9) for wild and captive‐bred anadromous salmon combined and of 173 (CI: 160.5–185.6) for all wild anadromous salmon and mature male parr (Figure 4). Thus, captive‐bred salmon increased the effective Nb by 28% and mature male parr increased it by 52%. Global estimate of Nb obtained from LDNe, including wild, captive‐bred, and mature male parr, was 211 (CI: 174–234).

**FIGURE 5:**
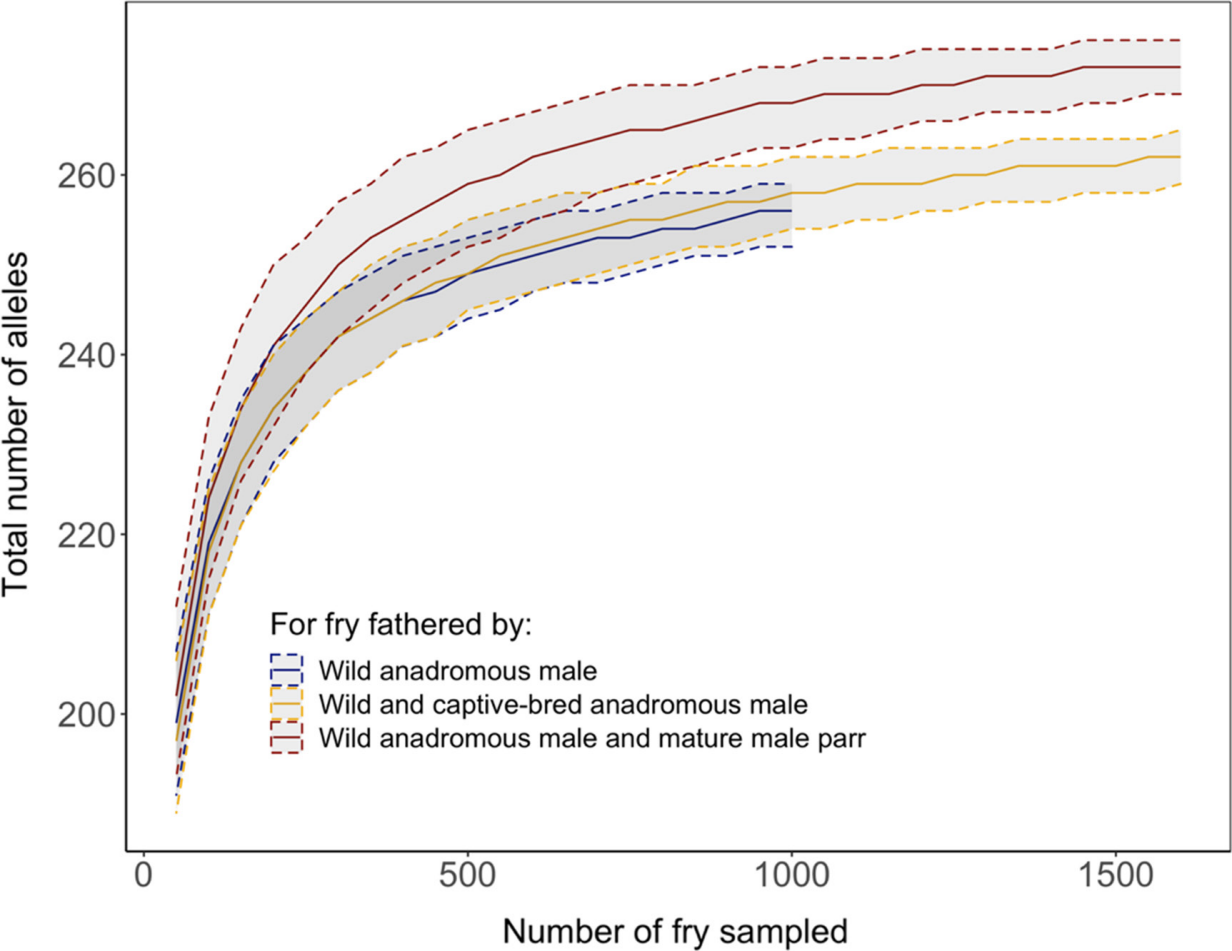
Loess regression of the mean value of the number of alleles over all genotyped microsatellite loci calculated for 50 to 1500 fry fathered by wild anadromous male, wild and captive‐bred anadromous male combined, and all wild anadromous male and mature male parr. Each estimate was bootstrapped 1000 times. Five to 95% interval distribution of the data is displayed around the mean value

Captive‐bred anadromous salmon and mature male parr both contributed to increasing the total number of alleles in the population relative to a scenario considering that only wild anadromous fish contributed to reproduction (Figure 5). However, only mature male parr significantly increased the allelic diversity. Indeed, fry produced by pairs of anadromous females and mature male parr had a higher number of alleles than fry produced by pairs of wild anadromous salmon and by wild and captive‐bred anadromous salmon. For instance, sampling 1000 offspring 1000 times in each dataset, the average number of alleles was 252 (CI: 252–259) for wild anadromous salmon, 258 (CI: 254–262) for wild and captive‐bred anadromous salmon, and 265 (CI: 265–272) for wild anadromous salmon and mature male parr.

## 4 DISCUSSION

In this study, we tested whether Atlantic salmon stocked at the parr stage have reduced reproductive success compared with their wild conspecifics and whether they contribute to increasing genetic diversity in the targeted population. Our results revealed that captive‐bred males and females displayed lower relative reproductive success and had fewer mates than their wild conspecifics. Moreover, we showed that both captive‐bred and mature male parr salmon contributed to increasing the effective number of breeders, but only mature male parr contributed to increasing allelic diversity compared with a scenario where only wild salmon contributed to reproduction. Below we underline the evolutionary and conservation implications of these results, which should contribute to guiding resource manager's decision on which life stages to stock for the restoration of Atlantic salmon populations.

### 4.1 Atlantic salmon mating system in the Rimouski River

#### 4.1.1 Female and male reproductive fitness

All females mated with multiple anadromous males and mature parr, which reflects the well‐documented male‐biased operational sex ratio in this species (Fleming et al., 1996; Petersson & Järvi, 2015; Weir, Breau, et al., 2010; Weir, Grant, et al., 2010; Weir et al., 2011, 2012). In such conditions, it has been shown that the display of aggressive behavior by MSW males has a low reproductive payoff because the high relative number of male competitors decreases the probability of successfully defend spawning females (Fleming & Gross, 1994). Hence, multiple male spawning event occurs, and sperm deposition is usually in order of dominance status (Fleming et al., 1996, 1997). Accordingly, Fleming and Einum (2011) showed that female reproductive success increased at a greater rate when mating with MSW mates than with 1SW and mature parr mates who adopt a sneaking strategy. As for females, there was high interindividual variance in reproductive success among males, which was best explained both by the number of mates and the number of years spent at sea (i.e., 1SW or MSW). This is in accordance with the fact that Atlantic salmon males do not provide any parental care, making their offspring production an increasing function of their number of mates.

#### 4.1.2 Mature male parr

We identified 432 mature parr male who fathered 30% of all offspring sampled. This is in broad accordance with previous studies, which reported proportions varying between 22 and 65% (Garcia‐Vazquez et al., 2001; Saura et al., 2008; Taggart et al., 2001; Weir, Breau, et al., 2010; Weir, Grant, et al., 2010). Compared with a previous study performed in Les Escoumins R. draining on the north shore of the St. Lawrence River in Québec, the mean reproductive success of mature parr was lower (1.7 vs 2.4; Richard et al., 2013). Because the aggressive behavior of anadromous males can restrain the participation of mature parr in reproduction (Jones & Hutchings, 2001; Tentelier et al., 2016), it could be that the spawning success of male parr was reduced by the larger proportion of anadromous males in our study (57% vs. 41% in Richard et al., 2013). However, this remains to be rigorously demonstrated.

### 4.2 Reduced fitness of captive‐bred Atlantic salmon stocked at the parr stage after one generation of captivity

The reduced RRS of captive‐bred Atlantic salmon corroborates results from Christie et al. (2014) who quantitatively synthesized the results of five studies that used genetic parentage analysis to estimate the fitness of first‐generation hatchery‐born adults and wild‐born adults spawning in the wild in various salmonid species. In 46 of 51 estimates, adults born in a hatchery had about 50% of the reproductive success of wild‐born adults. Similarly, in the one study on Atlantic salmon that was included in that review (Milot et al., 2013), the reproductive success of all captive‐bred salmon which were either stocked at the fry stage and the parr stage was nearly half that of wild fish (RRS = 0.55). Here, captive‐bred salmon at the parr stage had about 76% of the reproductive success of wild salmon.

Our ZINB model showed that captive breeding did not directly affect the number of offspring per mating event but rather the number of mating events. Thus, this suggests that captive‐bred females were able to find suitable spawning sites and display level of fecundity similar to that of wild counterparts. However, they mated with 1SW males less frequently than their wild counterparts. Fleming and Gross (1993) previously reported that captive‐bred females were less competitive in their ability to acquire a nesting territory during direct competition with wild females, which constrained them to build fewer nests (Fleming & Petersson, 2001). If captive‐bred females built fewer nests than wild females, MSW males could more readily defend their redds, which would decrease their probability of getting fertilized by 1SW males. Again, behavioral observation would be required to confirm this hypothesis.

As observed for females, reproductive success variation in males was not significantly explained by the origin when the number of mates and time spent at sea were included in the analysis. However, both MSW and 1SW captive‐bred males had fewer mates, which affected 1SW to a greater extent. A previous study on captive‐bred Atlantic salmon reported that progeny resulting from captive‐breeding practices was found to be more aggressive than wild‐born conspecifics (Blanchet et al., 2008) and that this behavioral syndrome can hold years after being released in nature (Fleming et al., 1997). An increase in aggressive behavior could potentially have led to reduced mating success in males, as previously observed by Fleming et al. (1997) using experimental settings.

Although we do not have behavioral data to support this hypothesis, a previous study on Atlantic salmon controlling for the genetic background revealed that the environmental effects of hatchery‐rearing up to the smolt stage (juvenile migrating at sea) may affect male behavior (Fleming et al., 1997). These authors found that while captive‐bred males had similar levels of aggression, they were involved in more prolonged aggressive encounters and incurred greater wounding and mortality than wild males. As a consequence, those males were less able to monopolize spawning and obtained 49% fewer mates than wild males. The difference between the number of mates was less striking in our study, but captive‐bred salmon were stocked in the Rimouski R. at the parr stage, which significantly reduced exposure to the hatchery environment and may have contributed to increased RRS compared with salmon stocked at the smolt stage (Milot et al., 2013).

Early environment is a deterministic factor that acts on later performance in fish (Jonsson, & Jonsson, 2009). Accordingly, mounting evidence points toward an epigenetic basis associated with the effect of hatchery environment on epigenetic reprogramming of the progeny produced in captivity (Christie et al., 2016; Gavery et al., 2018; Le Luyer et al., 2017; Leitwein et al., 2021; Wellband et al., 2021). Of particular interest is the study of Rodriguez‐Barreto et al. (2019), which showed that early‐life hatchery exposure changed the pattern of methylation of Atlantic salmon males and that corticotropin‐releasing factor receptor 1‐like was one of the differentially methylated regions in hatchery fish. This gene is involved in increased aggression and activity in another salmonid, the Arctic charr, *Salvelinus arcticus* (Backström et al., 2015). Backström et al.’s (2015) results also demonstrated that those changes were inheritable, which raises concern about the potential negative long‐term consequences of captive breeding. In our case, hatchery‐rearing clearly reduced the number of mates of both females and males and further study should investigate whether a causal relationship exists between epigenetic modifications and aggressiveness of captive‐bred Atlantic salmon.

### 4.3 Effectiveness of parr stocking to restore Atlantic salmon populations

An optimal stocking method should represent a trade‐off between the gain of survival obtained in captive settings, the survival of released juvenile and the mitigation of reproductive fitness difference between released individuals and their wild counterparts (Jonsson & Jonsson, 2009). In Atlantic salmon, stocking salmon at the parr stage could represent such trade‐off. First, it is the life stage that experiences the least mortality in the wild compared with younger life stages (Cunjack & Therrien, 1998). Hence, rearing in captivity until salmon reaches this life stage would allow managers to benefit from an increase in survival compared with what is observed in the wild. Second, the survival of captive‐bred individuals is usually higher when fish are released at a more developed stage in Atlantic salmon (Coghlan & Ringler, 2004; Johnson, 2004). Therefore, parr stockings could result in an increase in first‐summer survival compared with what has been previously documented in eye egg planting, unfed fry, and fry stocking (Caron et al., 1999; Coghlan & Ringler, 2004; Johnson, 2004).

While numerous studies recommend limiting the time spent in captivity (Araki & Schmid, 2010; Frankham, 2008; Fraser, 2008; Williams & Hoffman, 2009), our results indicate that salmon with fitness comparable to their wild counterpart can be obtained from stocking at the parr stage. Here, captive‐bred MSW females and males averaged ~80% of the reproductive success of their wild counterparts. Hence, compared with salmon stocked at the smolt stage, which had a RRS of 42% (CI: 20–74%) (Milot et al., 2013), salmon stocked at the parr stage performed better. Moreover, stocking at the parr stage produced salmon that have a RRS comparable to salmon stocked at the fry stage RRS of 71% (CI: 42–122%) (Milot et al., 2013). Although O’Sullivan et al. (2020) demonstrated that reduced reproductive success can reduce overall productivity of recipient natural populations in European Atlantic salmon, it is worth mentioning that captive‐bred salmon were released as smolt in the Burrishoole system. These salmon had 36% of the reproductive success of wild salmon on average; hence, releasing salmon at the parr stage could minimize impact on the recipient population's productivity. Taken together, these results suggest that parr stocking could represent a potential optimal trade‐off between the gain of survival obtained in captivity and the performance of the stocked fish in the wild.

### 4.4 Contribution of captive‐bred salmon and mature male parr to the effective Nb and their influence on genetic diversity

It is widely recognized that genetic diversity both within and between populations is important to conserve, and it is relevant to ask whether captive breeding programs may help maintain genetic diversity (Fraser, 2007). In this study, captive‐bred salmon significantly contributed to a 1.28‐fold increase in the effective number of breeders (Nb of 114 versus 146 without and with captive‐bred individuals, respectively). These results are in line with those of Araki, Waples, et al. (2007), Araki, Cooper, et al. (2007), which demonstrated that the use of local broodstock for stocking contributed to a 1.73‐fold increase in Nb over two generations. Hence, our study demonstrates that the design used to produce captive‐bred salmon in our system can contribute to increase both census size populations and the effective Nb in the recipient populations.

The contribution of male parr to reproduction led to a 1.52‐fold increase in Nb compared to a scenario without their reproductive contribution. This is in broad agreement with a similar study performed in another salmon population from the Malbaie R. on the north shore of the St. Lawrence River where mature male parr resulted in a 1.79‐fold increase in Nb compared to a Nb estimate without considering their contribution to reproduction (Perrier et al., 2014). Moreover, we demonstrate that the allelic richness found in pairs of wild anadromous female salmon and mature male parr was higher than that of wild and captive‐bred anadromous salmon. Hence, mature male parr most likely buffer against reduction of genetic diversity in populations with relatively small census size and as such could contribute to enhancing their evolutionary potential and ultimately the long‐term persistence of fisheries. Indeed, those males constitute a reservoir that can compensate for variations in the number of anadromous males returning from sea migration. This phenomenon, referred to as genetic compensation, is responsible for limiting the decrease in Ne when few anadromous male breeders survive until breeding (Araki, Cooper, et al., 2007; Araki, Waples, et al., 2007). Several studies now support their key role in maintaining the long‐term genetic diversity within salmon populations (Araki, Cooper, et al., 2007; Johnstone et al., 2013; Perrier et al., 2014) and in reducing inbreeding (Perrier et al., 2014).

### 4.5 Evolutionary and practical consequences of captive breeding on wild populations

This study provides an overview of the potential benefits and consequences of stocking Atlantic salmon at the parr for the restoration of threatened populations as and raises issues pertaining to the conservation of wild Atlantic salmon populations.

First, our results suggest that captive‐bred Atlantic salmon stocked at the parr stage have reduced relative reproductive fitness compared with wild salmon that could be in part due to a reduced number of mates in captive‐bred salmon. However, behavioral study of captive‐bred salmon would be necessary to confirm this result since mating success is determined by a combination of multiple factors, which were not taken into account in our study design. Nonetheless, Atlantic salmon stocked at the parr stage showed significantly improved relative reproductive success compared with salmon stocked at the smolt stage, which spends more time in captivity, and similar to salmon stocked at the fry stage, which spend less time in captivity (Milot et al., 2013). This information should guide resource managers’ decision on what best stocking practice to adopt for the restoration of Atlantic salmon populations.

Second, we showed that captive‐bred salmon significantly increase the effective Nb in the targeted population. This contribution of captive‐bred salmon is encouraging from a conservation perspective since contemporary captive‐breeding programs for Atlantic salmon generally aim to help populations retain 90% of their genetic diversity over 100 years by maintaining a Nb of at least 100 (Waples, 1990).

Finally, our results corroborate those of previous studies, showing that mature male parr contribute importantly to maintain genetic diversity. Anglers and managers generally pay little attention to the potential importance of mature male parr, either from a demographic or from a genetic standpoint. In fact, their presence has even been seen as having a negative effect on recruitment to fisheries and on harvestable biomass (Myers, 1984). However, our results suggest that the contribution of mature male parr to genetic diversity should always be considered and evaluated when managing Atlantic salmon populations.

## CONFLICT OF INTEREST

Louis Bernatchez is the Editor‐in‐Chief of Evolutionary Applications and a co‐author of this article. He was excluded from editorial decision‐making related to the acceptance and publication of this article. Editorial decision‐making was handled independently by Associate Editor Maren Wellenreuther to minimize bias.

## Supporting information

Appendix S1

## Data Availability

Raw data used for this study are available at the Dryad digital repository: https://datadryad.org/stash/share/c2Pv_77TPwZt‐U72_Ka5Lg1ZVTUQcpHz4E4v8DS9WJs.
