# Supplementary material for: Effects of stocking at the parr stage on the reproductive fitness and genetic diversity of a wild population of Atlantic salmon (Salmo salar L.)
Source: Evol Appl. 2022 Apr 18;15(5):838–52. doi: 10.1111/eva.13374 (PMC9108320; doi:10.1111/eva.13374)
Supplement: Supplementary file 1 — Appendix S1 [file EVA-15-838-s001.docx]

Supplementary file:


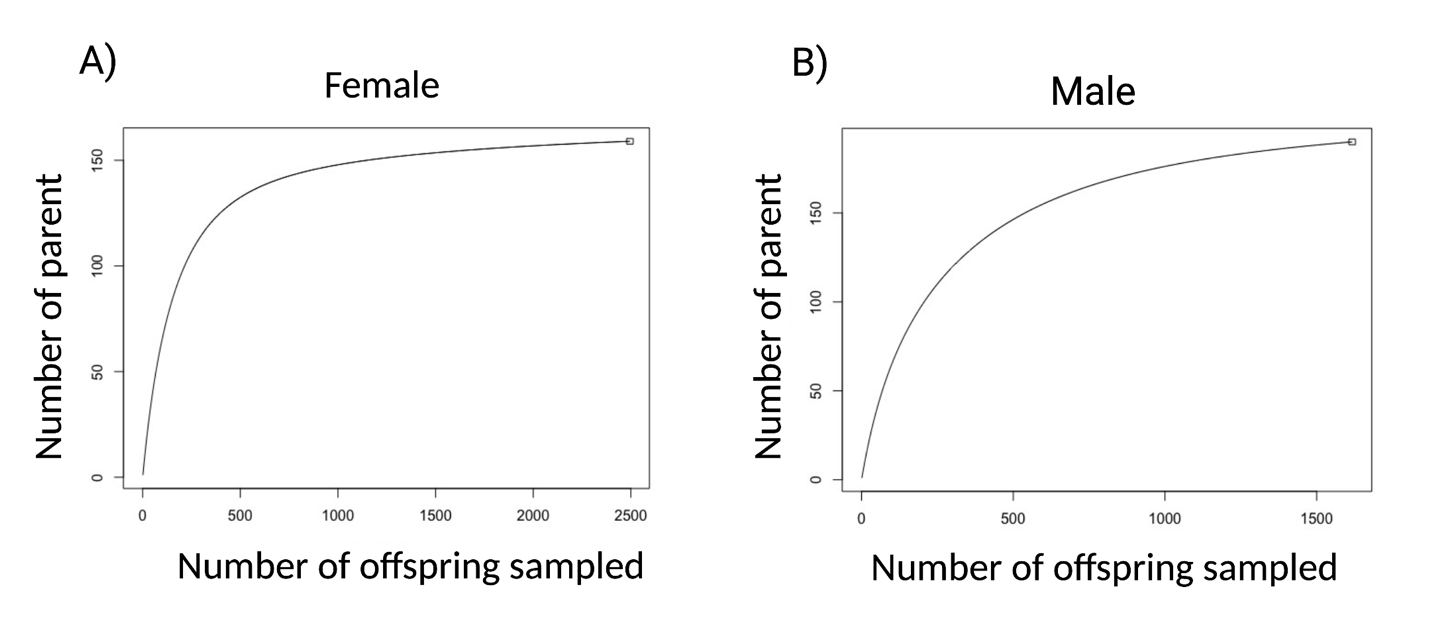


Figure S1: Rarefaction analysis curve for A) females and B) males. For both sexes, number of parents identified reach a plateau when increasing number of offspring sampled.


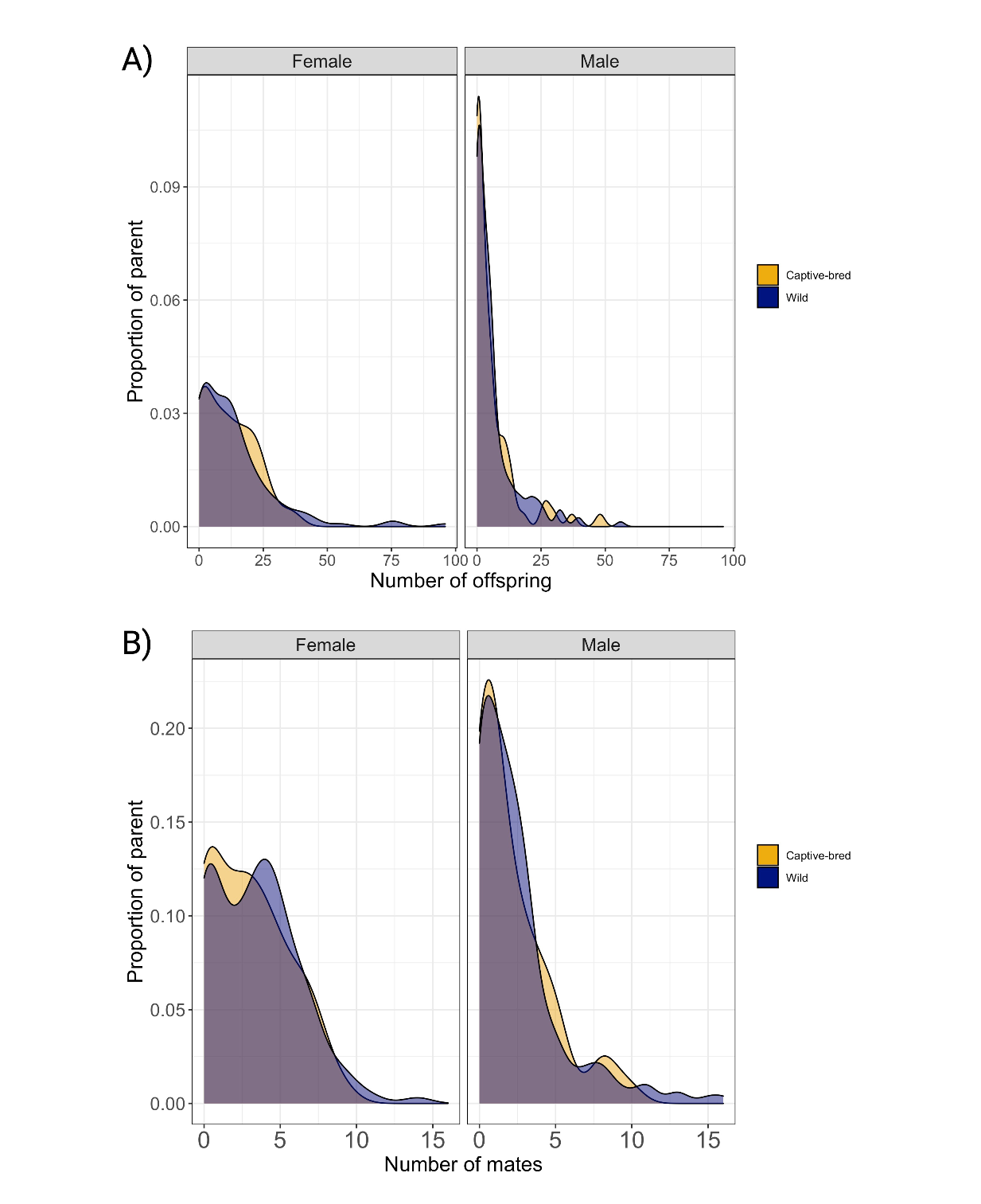


Figure S2: Density plots of the number of offspring produced by captive-bred vs. wild fish for females and males (1SW and MSW). Wild- and captive-bred-origin are depicted as blue and yellow, respectively.


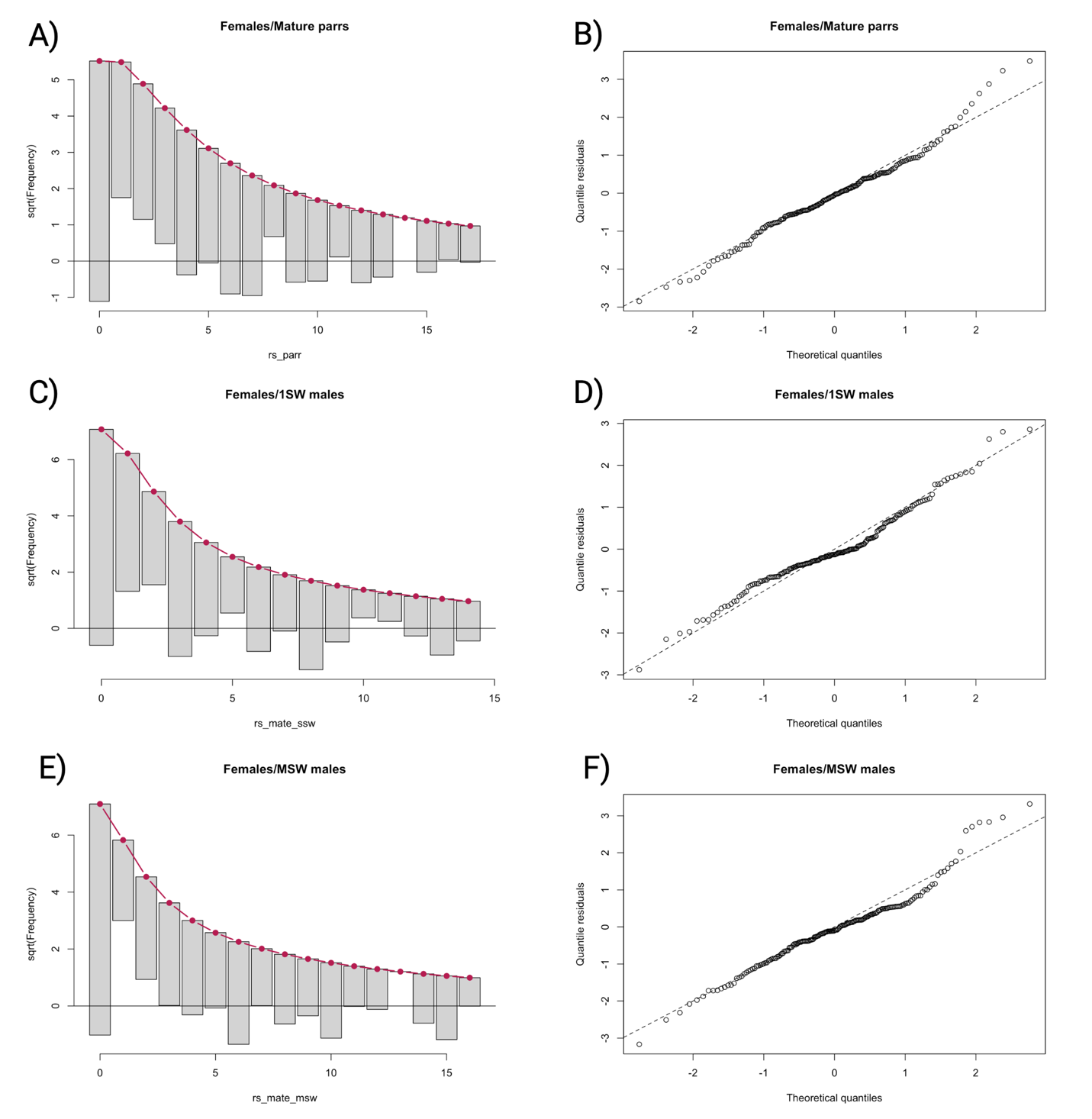


Figure S3: Rootogram (left) and quantile-quantile residuals (right) distribution for global models predicting reproductive success of females. A and B are the rootogram and quantile-quantile residuals distribution for models of factors predicting reproductive success of females when mating with mature parrs; C and D when mating with 1SW males, and E and F when mating with MSW males


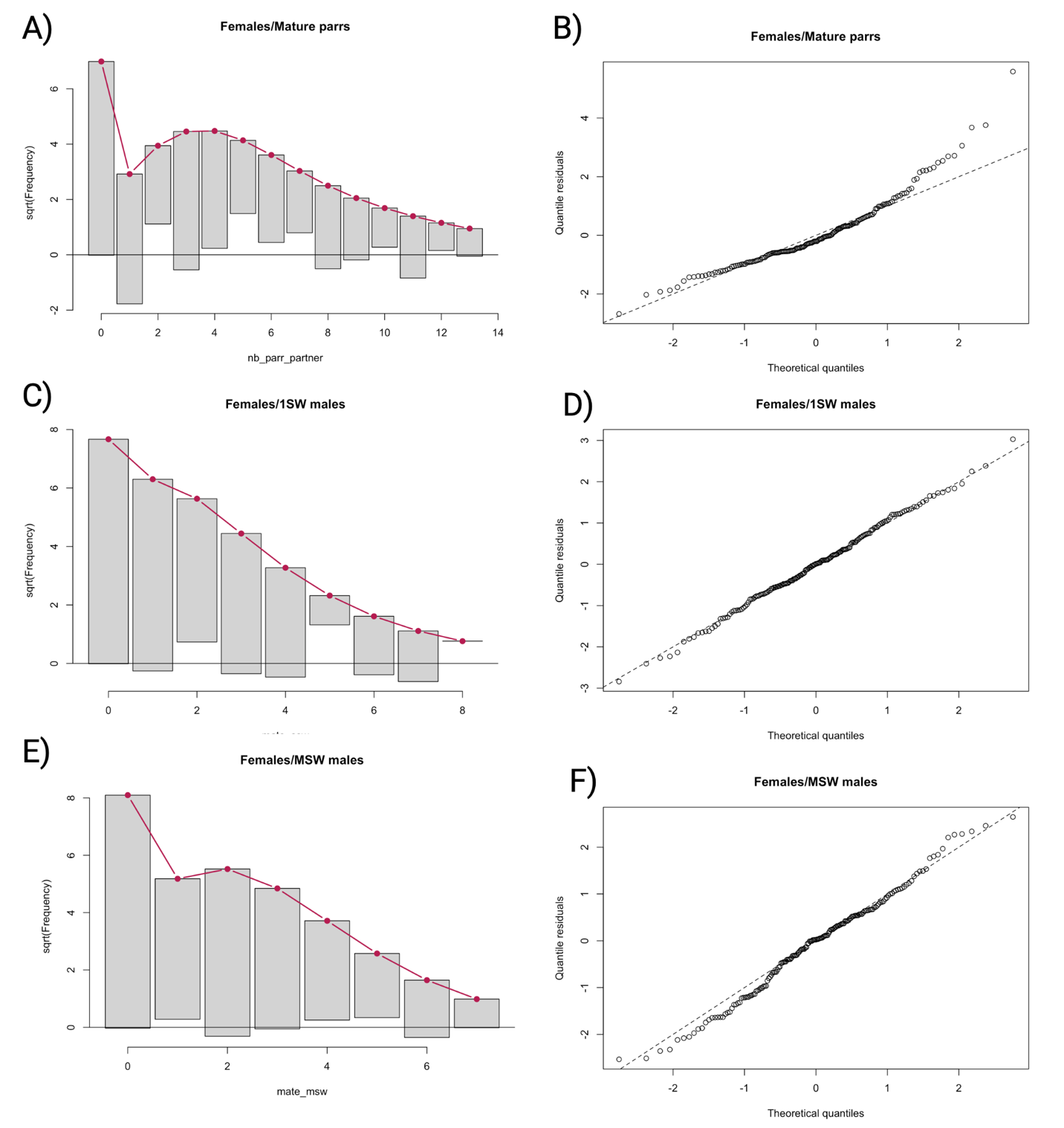


Figure S4: Rootogram (left) and quantile-quantile residuals (right) distribution for global models predicting number of mates of females. A and B are the rootogram and quantile-quantile residuals distribution for models of factors predicting number of mates of females when mating with mature parrs; C and D when mating with 1SW males, and E and F when mating with MSW males


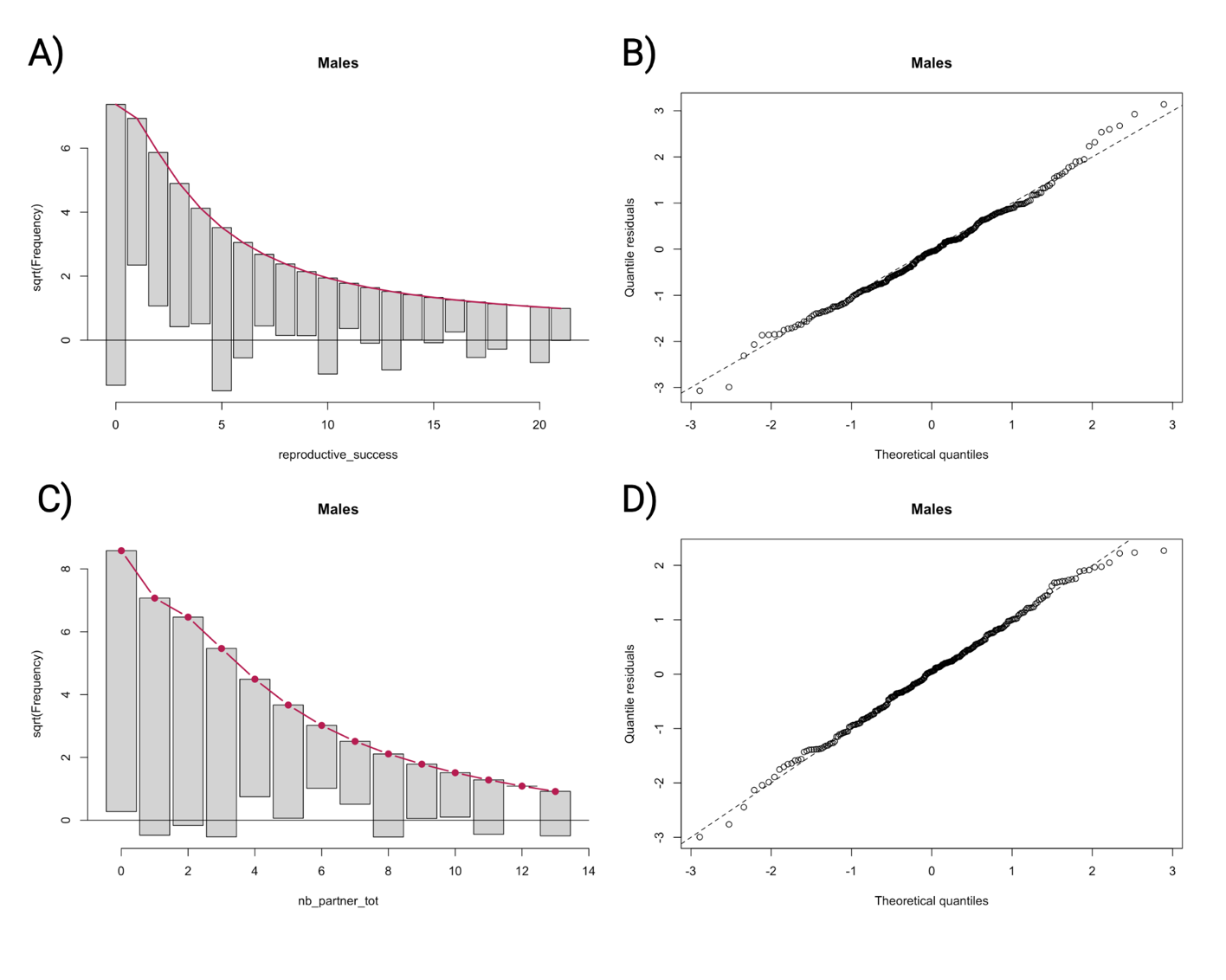


Figure S5: Rootogram (left) and quantile-quantile residuals (right) distribution for global models predicting number of mates of males. A and B are the rootogram and quantile-quantile residuals distribution for global models predicting reproductive success of males and C and D for global models predicting number of mates of males.

Table S1: Summary of observed heterozygosity (Ho), expected heterozygosity, and Fis per marker

| Locus name | Ho | He | Fis |
| --- | --- | --- | --- |
| NGS_SsaD486 | 0.7821 | 0.7803 | -0.0022 |
| NGS_SSsp2210 | 0.7393 | 0.7278 | -0.0159 |
| Ssa_1_7 | 0.5096 | 0.512 | 0.0047 |
| Ssa_1_8 | 0.1009 | 0.1106 | 0.0877 |
| Ssa_10_2 | 0.7058 | 0.7113 | 0.0078 |
| Ssa_11_2 | 0.565 | 0.5347 | -0.0567 |
| Ssa_11_3 | 0.7747 | 0.7904 | 0.0199 |
| Ssa_11_5 | 0.5544 | 0.5751 | 0.036 |
| Ssa_11_6 | 0.1517 | 0.1442 | -0.0524 |
| Ssa_12_5 | 0.4264 | 0.4362 | 0.0224 |
| Ssa_13_8 | 0.6333 | 0.6484 | 0.0233 |
| Ssa_14_2 | 0.3689 | 0.3676 | -0.0034 |
| Ssa_14_3 | 0.5342 | 0.5727 | 0.0673 |
| Ssa_14_6 | 0.54 | 0.5256 | -0.0274 |
| Ssa_15_1 | 0.5693 | 0.5597 | -0.0171 |
| Ssa_15_3 | 0.6802 | 0.6855 | 0.0077 |
| Ssa_19_1 | 0.8571 | 0.8401 | -0.0203 |
| Ssa_19_2 | 0.1876 | 0.1855 | -0.0115 |
| Ssa_19_3 | 0.6119 | 0.6062 | -0.0095 |
| Ssa_2_1 | 0.3774 | 0.3666 | -0.0295 |
| Ssa_2_2 | 0.5522 | 0.5568 | 0.0082 |
| Ssa_2_7 | 0.7015 | 0.695 | -0.0093 |
| Ssa_22_5 | 0.6812 | 0.6957 | 0.0207 |
| Ssa_23_2 | 0.7761 | 0.7368 | -0.0533 |
| Ssa_25_11 | 0.4212 | 0.4205 | -0.0015 |
| Ssa_25_2 | 0.7623 | 0.7624 | 1.00E-04 |
| Ssa_4_d44 | 0.6119 | 0.6408 | 0.0451 |
| Ssa_5_2 | 0.4691 | 0.4572 | -0.0259 |
| Ssa_6_2 | 0.5586 | 0.5491 | -0.0174 |
| Ssa_6_7 | 0.0045 | 0.0045 | -6.00E-04 |
| Ssa_7_11 | 0.5653 | 0.5708 | 0.0096 |
| Ssa_7_12 | 0.7015 | 0.6761 | -0.0376 |
| Ssa_9_3 | 0.5844 | 0.6025 | 0.03 |
| Ssa_9_8 | 0.6525 | 0.6544 | 0.003 |

Table S2. Models of factors predicting reproductive success and number of mates for females and males. All models were fit using a zero-inflated negative binomial model.

| **Dataset** | **Model structure** |
| --- | --- |
| Females | reproductive success = number of mates + length + origin |
|  | rumber of mates = length + origin |
| Males | reproductive success = number of mates * sea age + origin |
|  |  |
|  | number of mates = sea age +origin |
